# Improved Early Outcomes in Women Undergoing Aortic Valve Interventions

**DOI:** 10.3390/jcm12175749

**Published:** 2023-09-04

**Authors:** Pietro Giorgio Malvindi, Olimpia Bifulco, Paolo Berretta, Jacopo Alfonsi, Mariano Cefarelli, Carlo Zingaro, Filippo Capestro, Alessandro D’Alfonso, Marco Di Eusanio

**Affiliations:** Cardiac Surgery Unit, Lancisi Cardiovascular Center, Ospedali Riuniti delle Marche, Polytechnic University of Marche, 60126 Ancona, Italy

**Keywords:** aortic valve, aortic valve replacement, TAVI, women, gender

## Abstract

Surgical aortic valve replacement (SAVR) in female patients has been associated with higher mortality (up to 3.3–8.9%) and postoperative complication rates when compared with their male counterparts. In recent years, TAVI has been shown to provide a greater benefit than SAVR in women. We sought to assess the early outcomes of the contemporary aortic valve intervention practice (surgical and transcatheter) in patients referred to our cardiac surgery unit. The data of consecutive patients who underwent isolated aortic valve intervention for aortic valve stenosis during the 2018–2022 period were retrieved from our internal database. Several preoperative, intraoperative, and postoperative variables were analyzed, including the predicted risk of a prosthesis–patient mismatch. Nine hundred and fifty-five consecutive patients—514 women and 441 men—were included. Among them, 480 patients—276 female and 204 male—received a transcatheter procedure, and 475—238 women and 237 men—had conventional SAVR. The women were older and had higher EuroSCORE II, while the male patients presented a higher incidence of cardiovascular comorbidities. There was no difference in mortality or major postoperative complication rates after either the surgical or transcatheter procedures between the female and male populations. The availability and targeted use of different techniques and technologies have enabled the safe and effective treatment of female patients treated for severe symptomatic aortic valve stenosis with similar results when compared with their male counterparts.

## 1. Introduction

Aortic valve (AV) stenosis is the most common valve disorder due to an aging population [1]. Although a similar prevalence has been reported in men and women, different studies have shown a gender difference in terms of clinical presentation, treatment, and prognosis [2]. Women are inclined to underestimate cardiac disease, and this usually causes a late diagnosis and a delayed referral. Compared with the male population undergoing aortic valve surgery, women are more commonly in New York Heart Association (NYHA) class III–IV, and they are usually operated on at an older age, being more frail and at a higher risk in surgery [3]. Moreover, diastolic dysfunction, elevated pulmonary artery pressure, and a small aortic annulus are frequent in women with severe aortic valve stenosis [4] and account for higher surgical mortality and a lower benefit on symptomatic and prognostic grounds [5].

Transcatheter aortic valve implantation (TAVI) has provided an option for patients at medium/high risk [6,7,8] or in the presence of technical difficulties for a conventional SAVR and enabled the treatment of many patients who would have been inoperable or left untreated in the past. In these scenarios, TAVI has been associated with low rates of post-procedural complications, low in-hospital mortality, and satisfactory hemodynamics, and it has become an appealing solution for the treatment of aortic valve stenosis in women [5,9,10]. Nevertheless, female patients undergoing TAVI exhibit a higher rate of vascular injury and annular complications when compared with their male counterparts.

The combination of the peculiar clinical and pathophysiological features, anatomy characteristics, advanced age, and frailty typical of women presenting with symptomatic aortic valve stenosis poses several well-recognized therapeutic and technical challenges to both the surgical and TAVI approaches. At our cardiac surgery unit, we provide both surgical and transcatheter aortic valve interventions. At the beginning of our experience, TAVI was reserved for high-risk scenarios [11], but nowadays, based on satisfactory results in younger and lower-risk patients [12,13], we are very keen to also consider a transcatheter option in potential surgical candidates who might benefit from the technical and hemodynamic advantages provided via TAVI. Similarly, in case of predicted difficulties or expected suboptimal results from transcatheter interventions, a conventional aortic valve replacement through minimally invasive cardiac surgery associated with an enhanced recovery protocol is also routinely offered to elderly and frail patients.

This study reports our experience in aortic valve intervention—embedding both surgical and TAVI procedures performed by the same team of cardiac surgeons—and discusses the impact of this contemporary practice on early outcomes in female patients treated for severe symptomatic aortic valve stenosis.

## 2. Materials and Methods

### 2.1. Study Design and Ethical Approval

This is a single-center, retrospective analysis of prospectively collected data. All patients’ characteristics, intraoperative data, and periprocedural data were collected from the internal database of the Cardiac Surgery Unit at Lancisi Cardiovascular Centre—Polytechnic University of Marche–Ancona (Italy). The study was approved by the local ethics committee (CERM 2019 361), and informed consent was obtained in all cases.

### 2.2. Population

All consecutive patients who underwent a first-time isolated aortic valve intervention with biological prostheses (SAVR and TAVI) for aortic valve stenosis during the 2018–2022 period were included.

### 2.3. Definitions

Preoperative characteristics were defined according to EuroSCORE definitions [14]. Outcomes were coded according to the VARC-3 criteria [15] with the relevant events collected till day 30 postoperative.

The values for the effective orifice area (EOA) for each type of prosthesis implanted during the study period were retrieved from companies’ publications [16,17], observational studies [18,19,20], or administrative documents [21]. The indexed EOA (iEOA = EOA/body surface area) was calculated and used to define the degree of expected prosthesis–patient mismatch [22], which was classified as absent if iEOA > 0.85 cm^2^/m^2^, moderate for values between 0.65 cm^2^/m^2^ and 0.85 cm^2^/m^2^, and severe if iEOA < 0.65 cm^2^/m^2^.

### 2.4. Surgical Aortic Valve Replacement

In the surgical aortic valve replacement group, a minimally invasive approach including upper ministernotomy or right mini thoracotomy associated with an enhanced recovery protocol was our preferred strategy.

Ministernotomy was the most used access. It was performed through a J-shaped incision (4–5 cm) from the sternomanubrial joint to the 3rd or 4th intercostal space. Cardiopulmonary bypass (CBP) was established through conventional central cannulation, and the right pulmonary vein was cannulated for left ventricle venting.

Right mini thoracotomy (MT) surgery was performed through anterior right thoracotomy or trans-axillary access. The first was performed through a 4–5-cm skin incision at the 2nd and 3rd intercostal spaces, followed by costochondral cartilage dislocation. Trans-axillary access has been extensively described in previous publications and includes a 4–5 cm incision at the level of the 3rd intercostal space on the anterior axillary line [23]. In these cases, CPB was usually established by means of femoral cannulation.

The key features of our enhanced recovery after surgery approach, besides reduced access and chest trauma, include the routine use of normothermia during standard and minimally invasive extracorporeal circulation (MiECC), combined with ultra-fast-track anesthesia with on-table extubation, proactive pain management, physiotherapy starting on day 0 postoperative, and immediate (usually within two hours) patient–family contact [24,25].

### 2.5. Trans-Catheter Aortic Valve Implantation

TAVI procedures included either a transfemoral or a transapical approach.

At the beginning of our program [11], the procedures were performed under general anesthesia and with femoral cut-down. Afterwards, we implemented an awake procedure and the fully percutaneous management of the femoral access, and this approach represents our daily practice nowadays (>95% of cases).

We used two platforms: the Edwards balloon expandable (Sapien 3^®^ and Sapien 3 Ultra (Edwards Life-Sciences Corp., Irvine, CA, USA)) and the Medtronic self-expandable (Evolut™ R, Evolut ™ Pro, Evolut™ Pro+ (Medtronic, Minneapolis, MN, USA)) prostheses. The prosthesis size was chosen according to the recommendation charts supplied by the companies, based on preoperative CT scan measurements.

### 2.6. Echocardiographic Assessment

Preoperative, intraprocedural, and postoperative echocardiograms were performed with all patients. Left ventricle function, aortic valve area, peak gradients, and mean gradients were measured via the echocardiography core lab.

### 2.7. Statistical Analysis

Continuous variables are expressed as means ± SDs or medians and interquartile ranges (IQRs), while categorical variables are presented as numbers and percentages. Student *t* or Mann–Whitney *U* tests and a chi-square test were used to compare continuous or categorial variables, respectively. A post hoc analysis after Χ^2^ was performed to study the changing proportion of TAVI and surgical aortic valve replacement during the study period. The difference in EuroSCORE II and age throughout the study period was analyzed using the Kruskal–Wallis test.

Statistical significance was defined as *p* < 0.05.

The analysis was generated using Statistical Analysis Software (SAS), Version 3.8, SAS University Edition (SAS Institute Inc., Cary, NC, USA).

## 3. Results

### 3.1. Baseline Characteristics

During the study period, 955 consecutive patients—514 women and 441 men—underwent an isolated aortic valve intervention for aortic valve stenosis with a biological prosthesis. Four hundred and eighty patients—276 female and 204 male—received a transcatheter procedure, and 475—238 women and 237 men—had conventional SAVR. 

**Figure 1:**
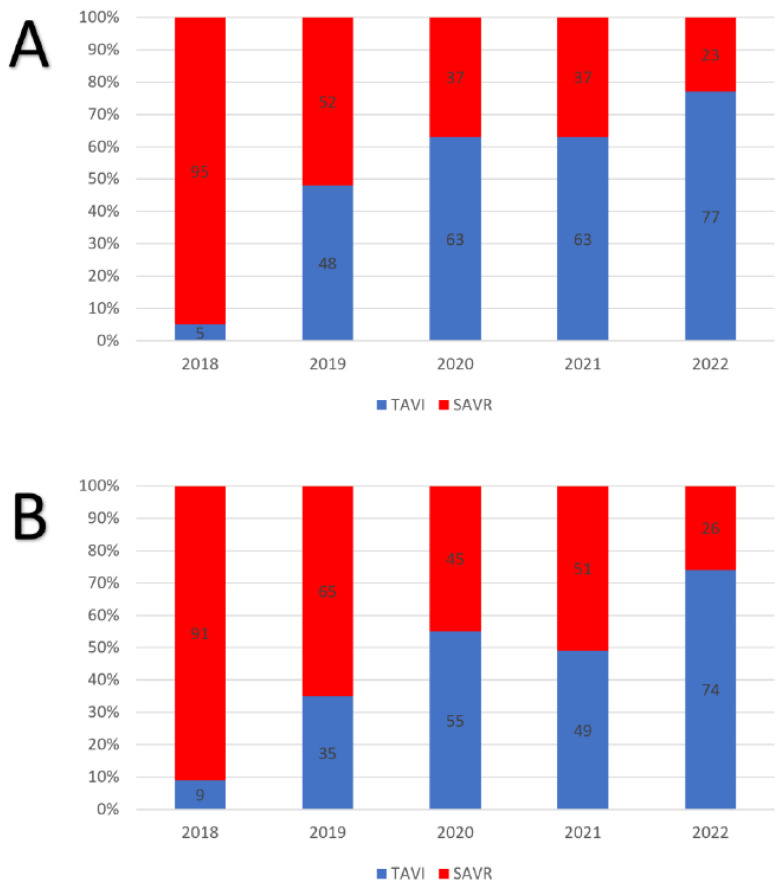
Temporal trend of TAVI and SAVR adoption in female ((**A**), *p* < 0.001) and male patients ((**B**), *p* < 0.001) during the study period.

There was a progressive increase in the use of transcatheter procedures throughout the study period in both the female and male populations with TAVI performed in more than 75% of the patients during the last year (*p* < 0.001; Figure 1). Since the start of our independent practice in transcatheter aortic valve procedures, the number of female patients treated for isolated aortic valve stenosis—TAVI and surgical aortic valve replacement—increased from 81 (year 2018) to a mean of 110 cases per year (+23%) and up to 115 cases per year (+25%) when excluding the cardiac surgery activity reduction due to the COVID-19 pandemic in 2020. Similarly, for men, there was an increase from 67 (2018) to 94 cases per year (+25%) and up to 99 cases per year (+32%) when excluding the year 2020.

During the study period, there was a significant decline in the mean age of female patients undergoing SAVR, from 74 years to 70 years (*p* = 0.009), with a concomitant reduction in EuroSCORE II values (*p* = 0.0015). Male SAVR patients exhibited a reduction in mean age throughout the study period, from 71 years to 68 years (*p* = 0.33), with no significant changes in EuroSCORE II (*p* = 0.73).

**Figure 2:**
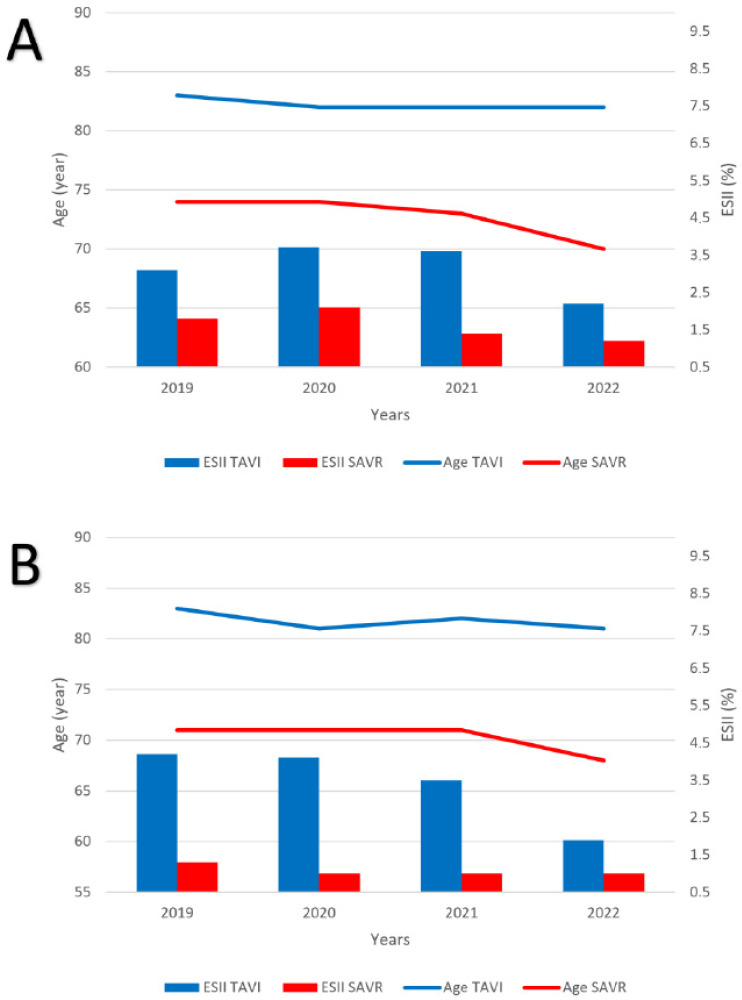
Temporal trend of EuroSCORE II and age according to transcatheter and surgical treatment in female (**A**) and male patients (**B**).

EuroSCORE II values reduced significantly during the study period for both female and male TAVI patients (*p* = 0.0015 and *p* = 0.009, respectively), but no changes were appreciated in patients’ mean age (*p* = 0.14 and *p* = 0.29, respectively). Figure 2 provides a graphical representation of the temporal trends in age and EuroSCORE II.

The female patients who came to our attention for aortic valve intervention were older, with a higher incidence of chronic kidney disease, and they faced a higher predicted operative risk than their male counterparts. On the contrary, men, despite lower EuroSCORE II values, presented with a higher incidence of preoperative cardiovascular comorbidities, including a history of CAD, a history of AMI, previous PCI or CABG, and peripheral vascular disease (Table 1). Similar differences were found in the subpopulations of female and male patients who underwent a TAVI procedure or a conventional surgical aortic valve replacement (Table 2).

The TAVI patients were older, at higher risk for surgery, and more symptomatic. The prevalence of smoking history, chronic obstructive pulmonary disease, rhythm disorders (including atrial fibrillation and previous pacemaker implantation), a history of CAD, and previous cerebrovascular accidents was higher in the TAVI population than in the SAVR population.

### 3.2. Operative Data

In the TAVI group, transapical and transfemoral approaches were performed, respectively, in 40 (14%) and 236 (86%) female patients and in 44 (22%) and 160 (78%) male patients. A balloon expandable prosthesis was used in 61% of cases in women and in 69% of cases in men. More than 75% of the female patients received a transcatheter prosthesis of [23,24,25,26] size, while more than 75% of men had a prosthesis size of [26,27,28,29] (Table 3).

Minimally invasive access was used in more than 80% of the surgical cases for both the populations of female and male patients. Three surgical prostheses were used, as follows: the Carpentier–Edwards Magna Ease aortic valve (Edwards Lifesciences, Irvine, CA, USA) in 53% of women and 89% of men, the Intuity Valve System (Edwards Life Sciences LLC, Irvine, CA, USA) in 25% of women and 2% of men, and the Trifecta bioprosthesis (Abbott Laboratories, Abbott Park, IL, USA) in 22% of women and 9% of men. About 75% of the female patients received a surgical prosthesis size of [21,22,23], while 90% of male patients had a prosthesis size ≥ 23. All operative data are presented in Table 4.

### 3.3. Postoperative Outcomes

The overall in-hospital mortality rate was 1% in the female population and 0.9% in the male population (*p* = 0.81), 1.4% and 1.5%, respectively, after TAVI (*p* = 0.99) and 0.4% in both groups after SAVR (*p* = 0.99). Neurological events were reported, respectively, in four female patients (0.8%) and five male patients (1.1%) (*p* = 0.57). After TAVI, we registered a 1.1% rate of postoperative stroke in women and a 2.5% rate in men (*p* = 0.29). One female patient (0.4%) suffered a neurologic event after SAVR vs. no event in the surgical male population (*p* = 0.99).

Furthermore, no differences were found in terms of respiratory failure, atrial fibrillation, vascular injury, or, in surgical cohorts, the length of postoperative intubation.

Women had a lower incidence of postoperative permanent pacemaker implantation and experienced a shorter stay in the ICU (*p* = 0.008). Table 5 and Table 6 summarize the data on early postoperative results.

### 3.4. Hemodynamics

Female patients had a higher preoperative peak and higher mean gradients across aortic valve and higher left ventricle ejection fraction than men in both the TAVI and SAVR cohorts. 

Based on patients’ characteristics and the size of the implanted prostheses, PPM was severe in 1.6% of cases—0.7% in the TAVI group and 2.5% in the SAVR group—in the female population. In the male population, PPM was severe in 0.4% of cases—0.5% in the TAVI group and 0.4% in the SAVR group.

At discharge, the postoperative mean and peak gradients were lower in the male population after both TAVI and SAVR.

A more than moderate degree of paravalvular regurgitation was described in 3.7% of female patients, 6.9% after TAVI, with no case reported after SAVR. Twelve male patients had a more than moderate degree of paravalvular regurgitation (2.7%), nine after TAVI (4.4%) and three after SAVR (1.3%). Table 7 and Table 8 detail the hemodynamic data.

## 4. Discussion

As the population ages, the burden of aortic valve stenosis is increasing. This pathology seems to be occurring with a similar prevalence in male and female patients, but several data suggest that severe aortic stenosis in women is undertreated. Surgical series from national registries have included many more cases of SAVR in men than in women (63% vs. 37%) [5]. Women receive a conservative treatment for a longer period than men, and when they present for aortic valve intervention, as furthermore highlighted by our data overall, the TAVI and surgical populations are usually older, with a higher operative risk profile, higher pulmonary pressure, and a smaller aortic valve area. Therefore, despite fewer previous cardiovascular events and a lower prevalence of coronary artery disease, previous acute myocardial infarction, and systemic vasculopathy, women experience worse early outcomes and longer hospital stays after SAVR compared with their male counterparts [5,10,26]. A worrisome in-hospital mortality rate, ranging from 3.3% up to 8.9%, has been reported in the literature for women undergoing isolated surgical aortic valve replacement, which is significantly higher than the 1.6–4.3% mortality rate registered for male patients from the same populations [5,10,26]. Female patients also experienced a higher rate of vascular complications, neurologic injury, transfusion, and nonhome discharge [5,27]. 

TAVI has emerged as an advantageous option for the treatment of aortic stenosis in women, for whom it provides a greater benefit than SAVR when compared with male patients [9,28]. Nevertheless, women undergoing TAVI have a non-negligible risk of major bleeding events and present a higher rate of non-femoral procedures, perioperative stroke, coronary obstruction, vascular injury, and pacemaker implantation compared to similar populations of male patients [28,29,30].

Our data did not confirm these findings. In the female SAVR population, we registered an in-hospital mortality rate and a postoperative stroke rate approaching 0% (0.4%), the need for CVVHD in 1.3% of cases, a median mechanical ventilation time of 5 h, and a median ICU stay of 24 h, with no difference when comparing these results with those of our male population. Similar satisfactory and comparable outcomes were found in female TAVI patients—hospital mortality at 1.4% and postoperative stroke at 1.1%—with a low occurrence of major vascular complications and severe paravalvular leak. The whole population of women who came to our attention for severe aortic stenosis—elderly (mean age: 80 years), symptomatic, and comorbid patients—received a safe treatment characterized by an in-hospital mortality rate of 1% and a stroke rate of 0.8%.

The main reason for these results lies in the careful selection among a multidisciplinary Heart Valve Team of the most appropriate option, surgical or transcatheter, considering the peculiar advantages and limitations of these techniques and the available technologies and devices.

At the beginning of our experience, the patients referred for a transcatheter procedure were older, frail, and at high risk for surgery [11]. The enrollment of these patients accounted for the increase in the mean EuroSCORE II value for the female patients we treated both surgically or by means of transcatheter interventions soon after the institution of our TAVI program. A similar trend has been described in previous experiences showing a greater referral of comorbid and higher-risk patients with a broadening of therapeutic options for patients who were deemed inoperable in the pre-TAVI era [31]. Over the years, as several RCTs and registries have provided the effectiveness and safety of TAVI in not only high-risk but also younger and low-risk patients [6,7,8,12], anatomical and technical factors, besides the usual clinical characteristics, have become important determinants of our interventional strategy to ensure the safest procedure with the best hemodynamics. 

As a result, in our recent practice, TAVI has progressively outperformed conventional SAVR, addressing a larger number of elderly patients. In this way, we have been able to provide interventional treatment to more female patients who fully benefited from the greater advantages that transcatheter procedures appear to confer on women over AVR [5,9,28] while avoiding challenging surgical procedures in the case of technical difficulties for a conventional surgical aortic valve replacement. Similarly, in the presence of hostile anatomy for a TAVI procedure with expected increased risks of vascular or annular complications, we have relied on our established surgical approach, contemplating reduced biological and psychological trauma, pursued through minimal surgical access, minimally invasive CPB, and anesthetic and physiotherapy protocols that promote enhanced recovery [24]. This approach has allowed us to close the gender gap that has historically seen female patients with aortic valve stenosis left largely undertreated [4,5] or operated on with higher rates of early complications and mortality [4,5,10,26].

Alongside the presentation at an increased age and with higher numbers of comorbidities, a smaller body size with concomitant smaller cardiac structures, smaller aortic annuli, and the implantation of 19- and 21-mm-size surgical prostheses [32,33] and ≤23-mm-size transcatheter valves [34] have been largely described in female patients undergoing aortic valve intervention, and they represent the main factors predisposing patients to a prosthesis–patient mismatch (PPM).

The prevention of PPM is important since it has been associated with worse hemodynamic function, less regression of left ventricular hypertrophy, and lower survival after SAVR [35,36]. Although its clinical impact after TAVI is still debated, there is increasing evidence underlying its negative impact on survival in patients with reduced left ventricular ejection fraction or in the presence of concomitant perivalvular regurgitation [37,38]. Moreover, the study of PPM after TAVI presents further limitations, lying in the low survival rate of high-risk, comorbid, and older patients, and the difficulties in obtaining echocardiographic measurements of EOA after transcatheter procedure [33,39,40,41]. We reported in women an expected severe PPM in 1.6% of cases—0.7% in the TAVI group and 2.5% in the surgical group—and an expected moderate and severe PPM in 26% of patients, values that are lower than those previously described in the literature on surgical and transcatheter series, ranging from 33% to 46% [33,36,42]. The availability of TAVI did not abolish the risk of PPM in our population, as a moderate PPM can be expected after the implantation of smaller-size transcatheter prostheses. Nevertheless, the risk of predicted PPM was markedly reduced via a careful selection of different techniques and devices, avoiding the use of bigger EOA mechanical prostheses that nowadays are not favored by our patients, as they represent a significant threat of bleeding complications in middle-aged and elderly patients [43,44,45].

Expertise and proficiency in both surgical and transcatheter approaches can provide a comprehensive understanding of the potential anatomical and technical challenges typical of female patients with aortic valve stenosis. Tailored use of these techniques—with the availability of suprannular stented valves for which we favor a continuous suture technique and sutureless prostheses, balloon-expandable or self-expandable valves [46,47], minimally invasive surgery, and femoral and non-femoral access for TAVI procedures—can not only reduce the burden of postoperative mortality and complications but also mitigate the risk of moderate PPM and minimize the occurrence of severe PPM in this high-risk population, thus potentially improving mid- and long-term outcomes [48]. These findings support an early-interventional attitude for women with severe symptomatic aortic valve stenosis and discourage a watchful waiting strategy—perhaps based on previous evidence of an elevated operative risk—that could ultimately lead these patients too late to an appropriate aortic valve procedure while they are in an advanced stage of heart failure, characterized by renal and pulmonary dysfunction, increased frailty, poor mobility, or chronic alteration of the vascular bed, which can severely affect the prognostic and symptomatic values associated with a timely aortic valve intervention [49,50]. 

Our study shares the usual limitations associated with observational retrospective studies, although all the data were retrieved from our internal database, which is prospectively completed by specialist doctors before the discharge of patients. A peculiar limitation of our analysis lies in the ongoing debate about the correct assessment of PPM following TAVI or SAVR using measured or predicted iEOA. We have used predicted iEOA [13,17,18,19,20], as it was derived from data from manufacturers’ charts or echocardiography studies. Predicted iEOA allows immediate use in clinical practice [39,51], although some reports have described a possible underestimation of the degree of PPM [52]. Measured PPM, on the other hand, depends largely on echocardiographic quality, the clinical profile of the patient (flow state), and the timing of ultrasound studies.

This study was not designed to provide any comparison of outcomes between the two populations of patients who had surgical or transcatheter aortic valve intervention. Instead, we aimed to highlight the importance of mastering both techniques and providing the most appropriate treatment, based on patients’ characteristics and anatomy, in order to increase the offering of therapeutic options and deliver the best possible results to the whole referral population.

No adjustment of preoperative characteristics was performed for the two populations of female and male patients since they differ a priori in disease presentation, natural history, and pathophysiology. Our study aims to provide an updated look at the early results in the treatment of women with aortic valve stenosis by comparing their outcomes with those observed in their male counterparts, fully considering their peculiar characteristics, which have historically been associated with poorer outcomes.

We acknowledge that lowering the threshold of TAVI with a broad implementation of transcatheter techniques in younger and lower-risk patients with a longer life expectancy warrants future research focusing on late survival, functional status, and valve durability, as recent studies showing satisfactory results on valve durability at 6–8 years are still based on longitudinal data from octogenarian populations with limited survival during follow-up [53,54].

## 5. Conclusions

Women presenting with severe symptomatic aortic valve stenosis can nowadays be treated without excess operative risk and can receive aortic valve intervention as safe and effective as those expected in male patients. The availability of different techniques and technologies has successfully addressed the clinical, anatomical, and technical difficulties usually encountered in female patients and translated into improved hospital mortality and postoperative complication rates with enhanced hemodynamics after both surgical and transcatheter procedures with similar early results when compared with male populations.

## Figures and Tables

**Table 1:** Preoperative characteristics.

Variable	Female (n = 514) N (%) or Median (IQR 1–3)	Male (n = 441) N (%) or Median (IQR 1–3)	*p* Value
Age (years)	80 (75–84)	77 (71–83)	<0.001
BMI (kg/m^2^)	26 (23.4–29.7)	27 (24.5–29.4)	0.015
BSA (m^2^)	1.7 (1.6–1.8)	1.9 (1.8–2.0)	<0.001
Euroscore II (%)	2 (1.4–3.4)	1.6 (1.0–2.6)	<0.001
NYHA class III–IV	279 (55)	218 (49)	0.14
Hypertension	427 (84)	368 (84)	0.80
Diabetes	121 (24)	114 (26)	0.49
Dyslipidemia	283 (56)	249 (56)	0.88
COPD	80 (16)	89 (20)	0.06
Atrial fibrillation	100 (19)	89 (20)	0.77
Pacemaker	14 (3)	29 (7)	0.046
eGFR < 50	231 (45)	165 (37)	0.018
Dialysis	4 (1)	9 (2)	0.10
History of CAD	122 (24)	172 (39)	<0.001
Previous AMI	22 (4)	48 (11)	<0.001
Previous PCI	54 (11)	98 (22)	<0.001
Previous CABG	10 (4)	33 (7)	0.034
Previous CVA	64 (13)	54 (12)	0.92
Peripheral arteriopathy	49 (10)	62 (14)	0.029

AMI: acute myocardial infarction. BMI: body mass index. BSA: body surface area. CABG: coronary artery bypass graft surgery. CAD: coronary artery disease. COPD: chronic obstructive pulmonary disease. CVA: cerebrovascular accident. IQR: interquartile range. LVEF: left ventricular ejection fraction. NYHA: New York Heart Association. PCI: percutaneous coronary intervention.

**Table 2:** Preoperative characteristics according to transcatheter and surgical treatment.

	TAVI			SAVR		
Variable	Female (n = 276) N (%) or Median (IQR 1–3)	Male (n = 204) N (%) or Median (IQR 1–3)	*p* Value	Female (n = 238) N (%) or Median (IQR 1–3)	Male (n = 237) N (%) or Median (IQR 1–3)	*p* Value
Age (years)	83 (80–86)	82 (79–85)	0.20	75 (70–79)	72 (67–76)	<0.001
BMI (kg/m^2^)	25.3 (22.5–28.7)	26.0 (24.1–29.1)	0.009	27.1 (24.3–30.5)	27.3 (24.8–29.6)	0.63
BSA (m^2^)	1.7 (1.6–1.8)	1.9 (1.8–2.0)	<0.001	1.7 (1.6–1.8)	1.9 (1.8–2.0)	<0.001
Euroscore II (%)	2.8 (1.8–4.1)	2.5 (1.6–4.7)	0.72	1.5 (1.2–2.4)	1.1 (0.8–1.6)	<0.001
NYHA class III–IV	167 (61)	129 (63)	0.54	112 (47)	89 (38)	0.04
Hypertension	226 (83)	166 (81)	0.51	201 (85)	202 (85)	0.90
Diabetes	66 (24)	61 (30)	0.19	55 (23)	53 (22)	0.90
Dyslipidemia	142 (52)	129 (63)	0.029	141 (59)	120 (51)	0.06
COPD	59 (22)	50 (25)	0.41	21 (9)	39 (17)	0.001
Atrial fibrillation	65 (24)	64 (32)	0.05	35 (15)	25 (11)	0.21
Pacemaker	11 (4)	22 (11)	0.003	3 (1.3)	7 (3)	0.22
eGFR < 50	161 (58)	115 (56)	0.67	70 (29)	50 (21)	0.037
Dialysis	4 (1.5)	6 (3)	0.33	0	3 (1)	0.12
History of CAD	80 (29)	114 (56)	<0.001	42 (18)	58 (25)	0.07
Previous AMI	12 (4)	30 (15)	<0.001	10 (4)	18 (8)	0.17
Previous PCI	45 (16)	68 (33)	<0.001	9 (4)	30 (13)	<0.001
Previous CABG	10 (4)	33 (16)	<0.001	-	-	-
Previous CVA	46 (17)	30 (15)	0.56	18 (8)	24 (10)	0.34
Peripheral arteriopathy	35 (14)	44 (22)	0.009	14 (6)	18 (8)	0.47

AMI: acute myocardial infarction. BMI: body mass index. BSA: body surface area. CABG: coronary artery bypass graft surgery. CAD: coronary artery disease. COPD: chronic obstructive pulmonary disease. CVA: cerebrovascular accident. IQR: interquartile range. LVEF: left ventricular ejection fraction. NYHA: New York Heart Association. PCI: percutaneous coronary intervention.

**Table 3:** Operative data on transcatheter procedures.

Variable	Female (n = 276)		Male (n = 204)		*p* Value
	N	%	N	%	
Type of prostheses					0.07
Edwards balloon-expandable	169	61	141	69	
Medtronic self-expandable	107	39	63	31	
Size of prosthesis					<0.001
20	9	3	0	0	
23	119	43	20	10	
26	99	36	86	42	
29	47	17	69	34	
34	2	1	29	14	
Access					0.043
Transapical access	40	14	44	22	
Transfemoral access	236	86	160	78	

**Table 4:** Operative data on surgical procedures.

Variable	Female (n = 238)		Male (n = 237)		*p* Value
	N	%	N	%	
Type of prosthesis					<0.001
Carpentier–Edwards Magna Ease	125	53	181	89	
Trifecta	53	22	18	9	
Intuity valve system	60	25	5	2	
Size of prosthesis					<0.001
19	54	23	3	1	
21	128	54	21	9	
23	50	21	110	46	
25	6	3	79	34	
27	0	0	17	7	
29	0	0	7	3	
Minimally invasive approach	196	82	192	81	0.71
Full sternotomy	42	18	45	19	
Cross-clamp time	53 (43–64)		57 (47–67)		0.017
CBP time	70 (60–82)		75 (62–88)		0.036

CBP: cardiopulmonary bypass.

**Table 5:** Postoperative outcomes.

Variable	Female (n = 514) N (%) or Median (IQR 1–3)	Male (n = 441) N (%) or Median (IQR 1–3)	*p* Value
In-hospital mortality	5 (1)	4 (0.9)	0.81
Stroke	4 (0.8)	5 (1.1)	0.57
Renal failure	38 (7.5)	48 (11)	0.06
CVVH	6 (1.2)	6 (1.4)	0.79
AMI	1 (0.2)	1 (0.2)	0.91
Respiratory insufficiency	10 (2)	8 (2)	0.88
Atrial fibrillation (in patients with preop SR)	97/414 (23)	95/352 (27)	0.46
Definitive pacemaker	52/500 (10)	68 (16.5)	0.006
Vascular complications			0.18
Major	5 (0.9)	2 (0.5)	
Minor	20 (3.9)	12 (2.7)	
Intubation time (hours)	5 (0–8)	5 (0–8)	0.28
ICU stay (hours)	24 (6–29)	24 (17–46)	0.008
Hospital stay (days)	6 (5–7)	6 (5–8)	0.24

AMI: acute myocardial infarction. CVVH: continuous veno-venous hemodialysis. ICU: intensive care unit. IQR: interquartile range. SR: sinus rhythm.

**Table 6:** Postoperative outcome according to TAVI and SAVR treatment.

	TAVI			SAVR		
Variable	Female (n = 276) N (%) or Median (IQR 1–3)	Male (n = 204) N (%) or Median (IQR 1–3)	*p* Value	Female (n = 238) N (%) or Median (IQR 1–3)	Male (n = 237) N (%) or Median (IQR 1–3)	*p* Value
In-hospital mortality	4 (1.4)	3 (1.5)	0.99	1 (0.4)	1 (0.4)	0.99
Stroke	3 (1.1)	5 (2.5)	0.29	1 (0.4)	0	0.99
Renal failure	24 (9)	29 (14)	0.06	14 (6)	19 (8)	0.37
CVVH	3 (1)	3 (1.5)	0.70	3 (1.3)	3 (1.3)	0.99
AMI	0	0	-	1 (0.4)	1 (0.4)	0.99
Respiratory insufficiency	5 (1.8)	4 (2)	0.99	5 (2.1)	4 (1.7)	0.99
Atrial fibrillation	21/211 (10)	16/139 (12)	0.70	76/203 (37)	79/213 (37)	0.99
Definitive pacemaker	46/265 (17)	56/182 (30)	<0.001	6 (3)	12/230 (5)	0.15
Vascular complications			0.18			-
Major	5 (2)	2 (1)		0	0	-
Minor	20 (7)	12 (6)		0	0	-
Intubation time (hours)	*	*		5 (0–8)	5 (0–8)	0.63
ICU stay (hours)	**	**		24 (23–48)	24 (23–48)	0.35
Hospital stay (days)	6 (5–7)	6 (5–8)	0.13	6 (5–8)	6 (5–7)	0.37

AMI: acute myocardial infarction. CVVH: continuous venovenous hemodialysis. ICU: intensive care unit. IQR: interquartile range. MOF: multiple organ failure. * > 95% of the patients received an awake procedure or were extubated on the table. ** TAVI patients are routinely transferred to a regular ward at the end of the procedure.

**Table 7:** Hemodynamic data.

Variable	Female (n = 514) N (%) or Median (IQR 1–3)	Male (n = 441) N (%) or Median (IQR 1–3)	*p* Value
Preoperative peak gradient (mmHg)	80 (70–99)	76 (67–90)	<0.001
Preoperative mean gradient (mmHg)	50 (41–60)	46 (41–55)	<0.001
AVA index (cm^2^)	0.4 (0.4–0.5)	0.4 (0.4–0.5)	0.28
Preoperative LVEF (%)	60 (55–65)	60 (50–63)	<0.001
Postoperative peak gradient (mmHg)	21 (16–27)	20 (16–25)	0.019
Postoperative mean gradient (mmHg)	11 (9–15)	10 (8–13)	0.007
Postoperative LVEF (%)	60 (55–65)	57 (50–60)	0.024
Paravalvular leak			0.40
Moderate	16 (3.1)	12 (2.7)	
Severe	3 (0.6)	0	
PPM	135 (26.3)	43 (9.8)	<0.001
Severe PPM	8 (1.6)	2 (0.4)	0.18

AVA: aortic valve area. EF: ejection fraction. IQR: interquartile range. LVEF: left ventricular ejection fraction. PPM: prosthesis–patient mismatch.

**Table 8:** Hemodynamic data according to TAVI and SAVR treatment.

	TAVI			SAVR		
Variable	Female (n = 276) N (%) or Median (IQR 1–3)	Male (n = 204) N (%) or Median (IQR 1–3)	*p* Value	Female (n = 238) N (%) or Median (IQR 1–3)	Male (n = 237) N (%) or Median (IQR 1–3)	*p* Value
Preoperative peak gradient (mmHg)	78 (68–97)	73 (64–85)	<0.001	84 (72–101)	80 (70–95)	0.05
Preoperative mean gradient (mmHg)	48 (41–60)	45 (40–52)	0.001	52 (42–63)	49 (41–59)	0.034
AVA index (cm^2^)	0.4 (0.4–0.6)	0.4 (0.4–0.5)	0.62	0.4 (0.4–0.5)	0.4 (0.4–0.5)	0.28
Preoperative LVEF (%)	60 (55–65)	56 (45–61)	<0.001	60 (58–65)	60 (55–65)	0.002
Postoperative peak gradient (mmHg)	20 (15–24)	19 (14–23)	0.09	23 (18–29)	20 (17–26)	0.016
Postoperative mean gradient (mmHg)	11 (8–13)	10 (7–12)	0.002	12 (10–16)	11 (9–15)	0.033
Postoperative LVEF (%)	60 (55–65)	55 (48–60)	<0.001	60 (55–65)	60 (55–63)	0.024
Paravalvular leak			0.47			0.25
Moderate	16 (5.8)	9 (4.4)		0	3 (1.3)	
Severe	3 (1.1)	0		0	0	
PPM	72 (26)	31 (15)	0.004	63 (27)	12 (5)	<0.001
Severe PPM	2 (0.7)	1 (0.5)	0.79	6 (2.5)	1 (0.4)	0.13

AVA: aortic valve area. EF: ejection fraction. IQR: interquartile range. LVEF: left ventricular ejection fraction. PPM: prosthesis–patient mismatch.

## Data Availability

Data are available on request due to restrictions.

## References

[1] Eveborn G.W., Schirmer H., Heggelund G., Lunde P., Rasmussen K. (2013). The evolving epidemiology of valvular aortic stenosis. the Tromsø study. Heart.

[2] Shan Y., Pellikka P.A. (2020). Aortic stenosis in women. Heart.

[3] Nau D.P., Ellis J.J., Kline-Rogers E.M., Mallya U., Eagle K.A., Erickson S.R. (2005). Gender and perceived severity of cardiac disease: Evidence that women are “tougher”. Am. J. Med..

[4] Tribouilloy C., Bohbot Y., Rusinaru D., Belkhir K., Diouf M., Altes A., Delpierre Q., Serbout S., Kubala M., Levy F. (2021). Excess Mortality and Undertreatment of Women with Severe Aortic Stenosis. J. Am. Heart Assoc..

[5] Chaker Z., Badhwar V., Alqahtani F., Aljohani S., Zack C.J., Holmes D.R., Rihal C.S., Alkhouli M. (2017). Sex Differences in the Utilization and Outcomes of Surgical Aortic Valve Replacement for Severe Aortic Stenosis. J. Am. Heart Assoc..

[6] Mack M.J., Leon M.B., Smith C.R., Miller D.C., Moses J.W., Tuzcu E.M., Webb J.G., Douglas P.S., Anderson W.N., Blackstone E.H. (2015). 5-year outcomes of transcatheter aortic valve replacement or surgical aortic valve replacement for high surgical risk patients with aortic stenosis (PARTNER 1): A randomised controlled trial. Lancet.

[7] Leon M.B., Smith C.R., Mack M.J., Makkar R.R., Svensson L.G., Kodali S.K., Thourani V.H., Tuzcu E.M., Miller D.C., Herrmann H.C. (2016). Transcatheter or Surgical Aortic-Valve Replacement in Intermediate-Risk Patients. N. Engl. J. Med..

[8] Toff W.D., Hildick-Smith D., Kovac J., Mullen M.J., Wendler O., Mansouri A., Rombach I., Abrams K.R., Conroy S.P., UK TAVI Trial Investigators (2022). Effect of Transcatheter Aortic Valve Implantation vs. Surgical Aortic Valve Replacement on All-Cause Mortality in Patients with Aortic Stenosis: A Randomized Clinical Trial. JAMA.

[9] Skelding K.A., Yakubov S.J., Kleiman N.S., Reardon M.J., Adams D.H., Huang J., Forrest J.K., Popma J.J. (2016). Transcatheter Aortic Valve Replacement Versus Surgery in Women at High Risk for Surgical Aortic Valve Replacement (from the CoreValve US High Risk Pivotal Trial). Am. J. Cardiol..

[10] Denegri A., Romano M., Petronio A.S., Angelillis M., Giannini C., Fiorina C., Branca L., Barbanti M., Costa G., Brambilla N. (2021). Gender Differences after Transcatheter Aortic Valve Replacement (TAVR): Insights from the Italian Clinical Service Project. J. Cardiovasc. Dev. Dis..

[11] Malvindi P.G., Berretta P., Capestro F., Bifulco O., Alfonsi J., Cefarelli M., Pierri M.D., Di Eusanio M. (2023). Results and insights after 413 TAVI procedures performed by cardiac surgeons on their own. Interdiscip. Cardiovasc. Thorac. Surg..

[12] Mack M.J., Leon M.B., Thourani V.H., Makkar R., Kodali S.K., Russo M., Kapadia S.R., Malaisrie S.C., Cohen D.J., Pibarot P. (2019). Transcatheter Aortic-Valve Replacement with a Balloon-Expandable Valve in Low-Risk Patients. N. Engl. J. Med..

[13] Popma J.J., Deeb G.M., Yakubov S.J., Mumtaz M., Gada H., O’Hair D., Bajwa T., Heiser J.C., Merhi W., Kleiman N.S. (2019). Transcatheter Aortic-Valve Replacement with a Self-Expanding Valve in Low-Risk Patients. N. Engl. J. Med..

[14] Nashef S.A., Roques F., Sharples L.D., Nilsson J., Smith C., Goldstone A.R., Lockowandt U. (2012). EuroSCORE II. Eur. J. Cardiothorac. Surg..

[15] Généreux P., Piazza N., Alu M.C., Nazif T., Hahn R.T., Pibarot P., Bax J.J., Leipsic J.A., Blanke P., VARC-3 Writing Committee (2021). Valve Academic Research Consortium 3: Updated Endpoint Definitions for Aortic Valve Clinical Research. J. Am. Coll. Cardiol..

[16] Hahn R.T., Leipsic J., Douglas P.S., Jaber W.A., Weissman N.J., Pibarot P., Blanke P., Oh J.K. (2019). Comprehensive Echocardiographic Assessment of Normal Transcatheter Valve Function. JACC Cardiovasc. Imaging.

[17] Mayr B., Burri M., Vitanova K., Prinzing A., Goppel G., Krane M., Lange R., Günzinger R. (2021). Serial echocardiographic evaluation of the Perimount Magna Ease prosthesis. J. Thorac. Dis..

[18] Barnhart G.R., Accola K.D., Grossi E.A., Woo Y.J., Mumtaz M.A., Sabik J.F., Slachman F.N., Patel H.J., Borger M.A., Garrett H.E. (2017). TRANSFORM (Multicenter Experience with Rapid Deployment Edwards INTUITY Valve System for Aortic Valve Replacement) US clinical trial: Performance of a rapid deployment aortic valve. J. Thorac. Cardiovasc. Surg..

[19] Lee H., Hwang H.Y., Sohn S.H., Choi J.W., Park J.B., Kim K.H., Kim K.B. (2020). Hemodynamic Performance of Pericardial Bioprostheses in the Aortic Position. Korean J. Thorac. Cardiovasc. Surg..

[20] Wyss T.R., Bigler M., Stalder M., Englberger L., Aymard T., Kadner A., Carrel T.P. (2010). Absence of prosthesis-patient mismatch with the new generation of Edwards stented aortic bioprosthesis. Interact. Cardiovasc. Thorac. Surg..

[21] https://www.accessdata.fda.gov/cdrh_docs/pdf10/P100029b.pdf.

[22] Rahimtoola S.H. (1978). The problem of valve prosthesis-patient mismatch. Circulation.

[23] https://www.minicardiacsurgery-univpm-research.com/video-gallery.

[24] Berretta P., De Angelis V., Alfonsi J., Pierri M.D., Malvindi P.G., Zahedi H.M., Munch C., Di Eusanio M. (2023). Enhanced recovery after minimally invasive heart valve surgery: Early and midterm outcomes. Int. J. Cardiol..

[25] Berretta P., Cefarelli M., Montecchiani L., Alfonsi J., Vessella W., Zahedi M.H., Carozza R., Munch C., Di Eusanio M. (2020). Minimally invasive versus standard extracorporeal circulation system in minimally invasive aortic valve surgery: A propensity score-matched study. Eur. J. Cardiothorac. Surg..

[26] Caponcello M.G., Banderas L.M., Ferrero C., Bramlage C., Thoenes M., Bramlage P. (2020). Gender differences in aortic valve replacement: Is surgical aortic valve replacement riskier and transcatheter aortic valve replacement safer in women than in men?. J. Thorac. Dis..

[27] Elhmidi Y., Piazza N., Mazzitelli D., Wottke M., Lange R., Bleiziffer S. (2014). Sex-related differences in 2197 patients undergoing isolated surgical aortic valve replacement. J. Card. Surg..

[28] Chandrasekhar J., Dangas G., Yu J., Vemulapalli S., Suchindran S., Vora A.N., Baber U., Mehran R., STS/ACC TVT Registry (2016). Sex-Based Differences in Outcomes with Transcatheter Aortic Valve Therapy: TVT Registry from 2011 to 2014. J. Am. Coll. Cardiol..

[29] Humphries K.H., Toggweiler S., Rodés-Cabau J., Nombela-Franco L., Dumont E., Wood D.A., Willson A.B., Binder R.K., Freeman M., Lee M.K. (2012). Sex differences in mortality after transcatheter aortic valve replacement for severe aortic stenosis. J. Am. Coll. Cardiol..

[30] O’Connor S.A., Morice M.C., Gilard M., Leon M.B., Webb J.G., Dvir D., Rodés-Cabau J., Tamburino C., Capodanno D., D’Ascenzo F. (2015). Revisiting Sex Equality with Transcatheter Aortic Valve Replacement Outcomes: A Collaborative, Patient-Level Meta-Analysis of 11,310 Patients. J. Am. Coll. Cardiol..

[31] Baran J., Podolec J., Tomala M.T., Nawrotek B., Niewiara Ł., Gackowski A., Przewłocki T., Żmudka K., Kabłak-Ziembicka A. (2018). Increased risk profile in the treatment of patients with symptomatic degenerative aortic valve stenosis over the last 10 years. Postep. Kardiol Interwencyjnej.

[32] Kapetanakis E.I., Athanasiou T., Mestres C.A., Nashef S.A., Aagaard J., Moritz A., Van Ingen G., Chronidou F., Palatianos G., Alivizatos P.A. (2008). Aortic valve replacement: Is there an implant size variation across Europe?. J. Heart Valve Dis..

[33] Franzen S.F., Huljebrant I.E., Konstantinov I.E., Nylander E., Olin C.L. (1996). Aortic valve replacement for aortic stenosis in patients with small aortic root. J. Heart Valve Dis..

[34] Panoulas V.F., Chandrasekhar J., Busi G., Ruparelia N., Zhang Z., Mehilli J., Sartori S., Lefèvre T., Presbitero P., Capranzano P. (2021). Prevalence, predictors, and outcomes of patient prosthesis mismatch in women undergoing TAVI for severe aortic stenosis: Insights from the WIN-TAVI registry. Catheter Cardiovasc. Interv..

[35] Sá M.P.B.O., de Carvalho M.M.B., Sobral Filho D.C., Cavalcanti L.R.P., Rayol S.D.C., Diniz R.G.S., Menezes A.M., Clavel M.A., Pibarot P., Lima R.C. (2019). Surgical aortic valve replacement and patient-prosthesis mismatch: A meta-analysis of 108 182 patients. Eur. J. Cardiothorac. Surg..

[36] Luthra S., Malvindi P.G., Olevano C., Zingale A., Salem H., Ohri S.K. (2021). Impact of valve size, predicted effective and indexed effective orifice area after aortic valve replacement. J. Card. Surg..

[37] Schofer N., Deuschl F., Rübsamen N., Skibowski J., Seiffert M., Voigtländer L., Schaefer A., Schneeberger Y., Schirmer J., Reichenspurner H. (2019). Prosthesis-patient mismatch after transcatheter aortic valve implantation: Prevalence and prognostic impact with respect to baseline left ventricular function. EuroIntervention.

[38] Pibarot P., Weissman N.J., Stewart W.J., Hahn R.T., Lindman B.R., McAndrew T., Kodali S.K., Mack M.J., Thourani V.H., Miller D.C. (2014). Incidence and sequelae of prosthesis-patient mismatch in transcatheter versus surgical valve replacement in high-risk patients with severe aortic stenosis: A PARTNER trial cohort—A analysis. J. Am. Coll. Cardiol..

[39] Takagi H., Umemoto T., ALICE (All-Literature Investigation of Cardiovascular Evidence) Group (2016). Prosthesis-Patient Mismatch After Transcatheter Aortic Valve Implantation. Ann. Thorac. Surg..

[40] Clavel M.A., Rodés-Cabau J., Dumont É., Bagur R., Bergeron S., De Larochellière R., Doyle D., Larose E., Dumesnil J.G., Pibarot P. (2011). Validation and characterization of transcatheter aortic valve effective orifice area measured by Doppler echocardiography. JACC Cardiovasc. Imaging.

[41] Deharo P., Leroux L., Theron A., Ferrara J., Vaillier A., Jaussaud N., Porto A., Morera P., Gariboldi V., Iung B. (2022). Long-Term Prognosis Value of Paravalvular Leak and Patient-Prosthesis Mismatch following Transcatheter Aortic Valve Implantation: Insight from the France-TAVI Registry. J. Clin. Med..

[42] Kolkailah A.A., Hirji S.A., Ejiofor J.I., Del Val F.R., Chowdhury R., McGurk S., Lee J., Kaneko T. (2020). Impact of Prosthesis Size and Prosthesis-Patient Mismatch on Outcomes in Younger Female Patients Undergoing Aortic Valve Replacement. Semin. Thorac. Cardiovasc. Surg..

[43] Goldstone A.B., Chiu P., Baiocchi M., Lingala B., Patrick W.L., Fischbein M.P., Woo Y.J. (2017). Mechanical or Biologic Prostheses for Aortic-Valve and Mitral-Valve Replacement. N. Engl. J. Med..

[44] Isaacs A.J., Shuhaiber J., Salemi A., Isom O.W., Sedrakyan A. (2015). National trends in utilization and in-hospital outcomes of mechanical versus bioprosthetic aortic valve replacements. J. Thorac. Cardiovasc. Surg..

[45] Malvindi P.G., Luthra S., Olevano C., Salem H., Kowalewski M., Ohri S. (2021). Aortic valve replacement with biological prosthesis in patients aged 50-69 years. Eur. J. Cardiothorac. Surg..

[46] Abdelghani M., Mankerious N., Allali A., Landt M., Kaur J., Sulimov D.S., Merten C., Sachse S., Mehilli J., Neumann F.J. (2018). Bioprosthetic Valve Performance After Transcatheter Aortic Valve Replacement with Self-Expanding Versus Balloon-Expandable Valves in Large Versus Small Aortic Valve Annuli: Insights from the CHOICE Trial and the CHOICE-Extend Registry. JACC Cardiovasc. Interv..

[47] Ferrara J., Theron A., Porto A., Morera P., Luporsi P., Jaussaud N., Gariboldi V., Collart F., Cuisset T., Deharo P. (2022). Prosthesis-Patient Mismatch in Small Aortic Annuli: Self-Expandable vs. Balloon-Expandable Transcatheter Aortic Valve Replacement. J. Clin. Med..

[48] Dismorr M., Glaser N., Franco-Cereceda A., Sartipy U. (2023). Effect of Prosthesis-Patient Mismatch on Long-Term Clinical Outcomes after Bioprosthetic Aortic Valve Replacement. J. Am. Coll. Cardiol..

[49] Geisler D., Rudziński P.N., Hasan W., Andreas M., Hasimbegovic E., Adlbrecht C., Winkler B., Weiss G., Strouhal A., Delle-Karth G. (2021). Identifying Patients without a Survival Benefit following Transfemoral and Transapical Transcatheter Aortic Valve Replacement. J. Clin. Med..

[50] Baran J., Kablak-Ziembicka A., Kleczynski P., Alfieri O., Niewiara Ł., Badacz R., Pieniazek P., Legutko J., Zmudka K., Przewlocki T. (2022). Association of Increased Vascular Stiffness with Cardiovascular Death and Heart Failure Episodes Following Intervention on Symptomatic Degenerative Aortic Stenosis. J. Clin. Med..

[51] Vogl B.J., El Shaer A., Crestanello J.A., Alkhouli M., Hatoum H. (2022). Flow dynamics in the sinus and downstream of third and fourth generation balloon expandable transcatheter aortic valves. J. Mech. Behav. Biomed. Mater..

[52] Ternacle J., Guimaraes L., Vincent F., Côté N., Côté M., Lachance D., Clavel M.A., Abbas A.E., Pibarot P., Rodés-Cabau J. (2021). Reclassification of prosthesis-patient mismatch after transcatheter aortic valve replacement using predicted vs. measured indexed effective orifice area. Eur. Heart J. Cardiovasc. Imaging.

[53] Jørgensen T.H., Thyregod H.G.H., Ihlemann N., Nissen H., Petursson P., Kjeldsen B.J., Steinbrüchel D.A., Olsen P.S., Søndergaard L. (2021). Eight-year outcomes for patients with aortic valve stenosis at low surgical risk randomized to transcatheter vs. surgical aortic valve replacement. Eur. Heart J..

[54] Ali N., Hildick-Smith D., Parker J., Malkin C.J., Cunnington M.S., Gurung S., Mailey J., MacCarthy P.A., Bharucha A., Brecker S.J. (2023). Long-term durability of self-expanding and balloon-expandable transcatheter aortic valve prostheses: UK TAVI registry. Catheter. Cardiovasc. Interv..

